# Whole-body magnetic resonance imaging (WBMRI) versus whole-body computed tomography (WBCT) for myeloma imaging and staging

**DOI:** 10.1007/s00256-021-03799-4

**Published:** 2021-05-24

**Authors:** Karla M. Treitl, Jens Ricke, Andrea Baur-Melnyk

**Affiliations:** Department of Radiology, University Hospital, LMU Munich, Marchioninistr. 15, 81377 Munich, Germany

**Keywords:** Plasma cell disorders, Multiple myeloma, Computed tomography, Magnetic resonance imaging, Whole-body imaging

## Abstract

Myeloma-associated bone disease (MBD) develops in about 80–90% of patients and severely affects their quality of life, as it accounts for the majority of mortality and morbidity. Imaging in multiple myeloma (MM) and MBD is of utmost importance in order to detect bone and bone marrow lesions as well as extraosseous soft-tissue masses and complications before the initiation of treatment. It is required for determination of the stage of disease and aids in the assessment of treatment response. Whole-body low-dose computed tomography (WBLDCT) is the key modality to establish the initial diagnosis of MM and is now recommended as reference standard procedure for the detection of lytic destruction in MBD. In contrast, whole-body magnetic resonance imaging (WBMRI) has higher sensitivity for the detection of focal and diffuse plasma cell infiltration patterns of the bone marrow and identifies them prior to osteolytic destruction. It is recommended for the evaluation of spinal and vertebral lesions, while functional, diffusion-weighted MRI (DWI-MRI) is a promising tool for the assessment of treatment response. This review addresses the current improvements and limitations of WBCT and WBMRI for diagnosis and staging in MM, underlining the fact that both modalities offer complementary information. It further summarizes the corresponding radiological findings and novel technological aspects of both modalities.

## Introduction

Plasma cell disorders (PCD) are a heterogenous group of monoclonal malignancies that evolve from terminally differentiated plasma cells and are classified according to the International Myeloma Working Group (IMWG) criteria [1]. Multiple myeloma (MM) accounts for 1% of all cancers and 10% of all hematological diseases [2]. In patients suffering from MM, the uncontrolled clonal proliferation of terminally differentiated B cells can be verified by bone marrow aspiration. It is associated with specific end-organ damage such as hypercalcemia, renal insufficiency, anemia or destructive bone lesions (CRAB criteria) [3, 4]. Alternatively, specific biomarkers, including a serum free light-chain ratio ≥ 100 or ≥ 60% of plasma cells in the bone marrow, indicate active MM, as well as magnetic resonance imaging (MRI) scans that reveal more than one focal bone lesion at least 5 mm in diameter [5]. Thus, MR imaging is for the first time a specific biomarker for the diagnosis of MM.

Myeloma-associated bone disease (MBD) is the most frequent manifestation of MM and accounts for the majority of mortality and morbidity associated with this PCD. The pathophysiological cause is a dysregulation of osteoblastic and osteoclastic interactions, leading to increased bone resorption without compensating bone formation, due to suppressed osteoblastic activity and upregulated osteoclastic activity [5]. It is characterized by the presence of osteolytic bone lesions on computed tomography (CT) [1, 5, 6]. Actually, 90% of patients will develop destructive bone lesions, while 80% will even suffer from a pathological fracture during the progression of their disease [7–9]. Therefore, MBD imaging is essential in the diagnosis, prognosis and follow-up of MM.

Imaging can further confirm the transition of a preliminary or intermediate stage of PCD, so-called monoclonal gammopathy of undetermined significance (MGUS) or smoldering multiple myeloma (SMM), to active MM by detecting osteolytic destruction. Furthermore, it is essential to differentiate solitary plasmacytoma from MM by [^18^F]-fluorodeoxyglucose positron emission tomography-CT ([^18^F]-FDG PET/CT) or MRI. MRI is the major diagnostic tool for identifying extraosseous manifestations and complications, such as pathological fractures and spinal cord compression.

The aim of this article is to review and compare whole-body CT (WBCT) and whole-body MRI (WBMRI) for diagnosis and staging in MM and to summarize the corresponding radiological findings.

## X-ray imaging (XR)

Conventional radiography (XR) underestimates the stage of disease and misses myeloma-related bone lesions in up to 70% of cases when compared to cross-sectional imaging techniques, as lesions become detectable on XR only after a decrease of at least 30–50% of bone mineral density [10, 11]. Therefore, cross-sectional and functional imaging techniques have replaced the whole-body radiographic skeletal (WBXR) survey as the gold standard for the radiological screening and diagnosis of MM. Both WBCT and WBMRI can diagnose intra- and extramedullary manifestations of MM with greater sensitivity and detect disease progression or relapse much earlier [12, 13]. They further provide prognostic information and are essential for the evaluation of therapeutic efficacy during staging and follow-up examinations [2, 14].

## Whole-body computed tomography (WBCT)

### Modality overview

As a cross-sectional imaging technique, whole-body computed tomography (WBCT) provides three-dimensional information and is able to display bone abnormalities associated with MM. WBCT is much more sensitive for detecting osteolytic lesions than WBXR because of the high contrast between cortical and cancellous bone tissue [15]. Since 2014, the guidelines of the IMWG state that the detection of one or more osteolytic bone lesions at least 5 mm in size on CT, including whole-body low-dose CT (WBLDCT) or PET/CT, meets the criteria for MBD [6]. In consequence, according to the European Myeloma Network and the European Society for Medical Oncology and the IMWG, WBLDCT is recommended as the initial reference standard procedure and the modality of choice to detect osteolytic MBD [16, 17]. Almost every MM patient receives a WBLDCT as baseline and follow-up staging examination [6]. [^18^F]-FDG PET/CT is a valuable alternative that is able to detect lytic bone lesions and extraosseous masses, while providing information based on the [^18^F]-FDG activity as well [18].

The IMWG guidelines further emphasize to need to avoid over-interpretation of small lucencies with a diameter below the threshold of 5 mm on CT images, and recommend re-examination of these particular patients after 3–6 months. Important differential diagnoses for hypodense trabecular bone lesions are benign fat-containing processes, which can be identified as false-positive lesions by CT density measurements. The most frequent examples are focal yellow bone marrow deposits, small hemangiomas and Modic type 2 degenerative vertebral endplate changes. Alternatively, an additional MRI examination or a biopsy of the specific lesions should be taken into consideration, if possible, in order to appropriately characterize them before establishing the diagnosis of MM and initiating systemic therapy. As MM is defined either by the percentage of bone marrow plasma cells ≥10% or biopsy-proven histological evidence of bony or extramedullary plasmacytoma, follow-up imaging is required if these criteria do not apply [14].

In patients with suspected SMM, the IMWG recommends at least one type of advanced imaging (PET/CT, WBLDCT, MRI of the pelvis and spine or WBMRI), depending on the availability and clinical situation, in order to distinguish SMM from active MM. Follow-up examinations are indicated in cases with changes in the clinical course or changing M-protein levels. High-risk patients in particular should receive periodic staging imaging to exclude asymptomatic progression [19]. Although patients with SMM may remain stable over extended periods of time, the progression rate to active MM is reported as 10% per year for the first 5 years and 3% per year for the following 5 years [20, 21].

WBLDCT further reliably excludes osteolysis in patients with MGUS and thereby completes the diagnostic hematological results in these cases. It also proved to be very sensitive in the depiction of newly developed lytic lesions in follow-up examinations of patients suffering from MGUS, who have an overall 1% risk of developing active MM [20, 22]. Risk factors for the progression of MGUS to active MM are M-protein levels of 1.5 g/dl or more and an abnormal free light-chain ratio in patients with non-IgM MGUS. However, the progression rate differs considerably between patients with no, one or two risk factors, and ranges from 7% to 20% and even 30% over 20 years [23]. Therefore, the IMWG recommends WBLDCT only in patients with high-risk MGUS.

In addition, CT is necessary for the planning of surgery in order to evaluate bony structure. Patients with osteoporosis need cement-augmented screw fixations, as osteolysis in the pedicles prohibits pedicle screws in this location. Another benefit of CT is its assistance in percutaneous biopsy for suspected lesions, in particular as the plasma cell percentage differs significantly between random bone marrow biopsy specimens and CT-guided samples, especially in focal disease [24, 25]. This in turn influences the final diagnosis, the staging of the disease and consequent treatment strategy.

### Imaging findings in WBLDCT

**Fig. 1:**
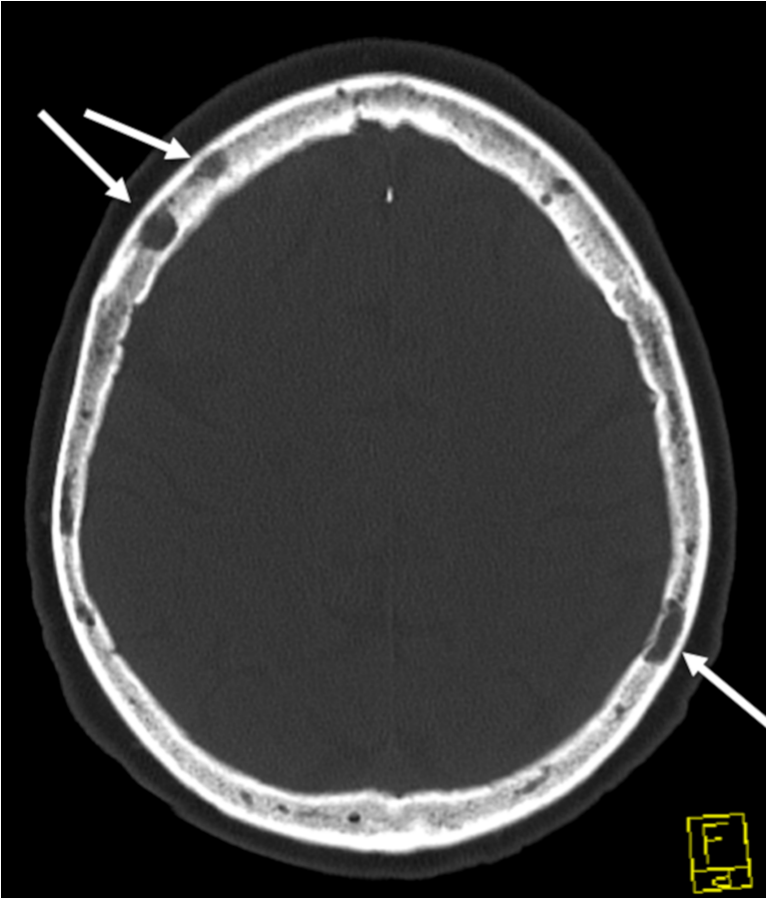
Axial view of a CT scan of the head in the bone window. The white arrows mark typical punched-out osteolytic lesions in the skull of a 48-year-old female patient with multiple myeloma

Lytic, well-circumscribed areas with non-sclerotic margins, producing a “punched-out” aspect, are regarded as typical and the most common manifestation of osteolytic lesions in MBD. The non-sclerotic halo is in contrast to most other bone metastases and indicates the decrease in osteoblastic activity (Fig. 1). They are primarily located in the trabecular bone of the axial skeleton and the pelvis, less frequently in the long bones of the upper and lower extremities [8, 14]. On WBLDCT, they are easily distinguished against normally mineralized trabecular bone, but may be difficult to distinguish in patients with advanced osteoporosis and rarefied trabeculae. Fat-containing, hypodense, benign lesions of the trabecular bone, such as typical hemangiomas, need to be discriminated from osteolytic lesions by density measurements. However, the detection of fat excludes osteolysis only in untreated patients, because treated lesions may feature a partial or total fatty replacement [17].

WBLDCT can further identify focal plasma cell infiltrates within the bone marrow of the appendicular skeleton, where they can be discriminated against fatty hypodense marrow (−30 HU to −100 HU), due to a higher density (average 55 HU) [14, 26]. A prospective study including 172 patients suffering from MGUS, SMM or active MM revealed that the percentage of medullary abnormalities and their CT density increased with increasing stage of disease. The authors consequently concluded that the CT density of focal medullary lesions of the appendicular skeleton correlates with disease progression and has prognostic value for the course of the disease [27].

Two different patterns of lytic bone lesions in MM were recently identified on WBLDCT [28]. The first pattern, with negative CT density values, had low ^18^[F]-FDG-uptake on PET/CT and a low apparent diffusion coefficient (ADC) on MRI, which is consistent with fatty marrow. The second pattern, with positive CT density values, had higher FDG-uptake on PET/CT and a higher ADC value on MRI, which is consistent with an infiltrative pattern. However, histology documented a plasma cell infiltration in both types of lesions and revealed that neoplastic plasma cells were scattered among adipocytes in the first pattern. Moreover, both patterns were simultaneously found in patients with symptomatic bone disease indicating active MM. Additionally, some of the HU-negative lesions became HU-positive in follow-up WBLDCT and developed an FDG-uptake and a pathological ADC. The investigators concluded that the initially hypodense, fat-rich lesions represent a preliminary stage that advances into hyperdense, infiltrative lesions with a higher concentration of plasma cells. However, the current IMWG criteria do not incorporate the CT density of myeloma lesions.

Endosteal scalloping and cortical disruption are typical findings when osteolytic lesions extend into the cortex of bones. When the cortex is disrupted, they can show soft-tissue masses. In some rare cases, MM may present osteosclerotic lesions, especially in POEMS (polyneuropathy, organomegaly, endocrinopathy, monoclonal protein, skin changes) syndrome, which is addressed in a separate article in this special issue.

A diffuse infiltration pattern in MM presents as diffuse osteoporosis and is hard to discriminate from senile or postmenopausal osteoporosis on WBLDCT. Age-related osteoporosis is associated with increasing fat content of the bone marrow, while a diffuse infiltration causes hypercellularity. MRI is the modality of choice to depict the hypercellularity in these cases using enhanced and unenhanced T1-weighted (T1w) spin-echo (SE) sequences [29, 30]. Dual-energy CT (DECT) may overcome this problem by generating virtual non-calcium (VNCa) images. However, the technique is not yet available everywhere.

In addition to bone imaging, WBLDCT is able to identify extraosseous or extramedullary myeloma, which can either appear as para-osseous soft-tissue masses adjacent to bones or may atypically manifest as pulmonary, intra-abdominal or intracranial tumoral lesions. The most frequently involved anatomical structures are the head and neck region, lymph nodes, the pleura, the spleen and the liver [20, 31, 32]. The sensitivity for the detection of extraosseous myeloma on CT images is increased by the intravenous administration of contrast. The presence of extraosseous disease and an extramedullary relapse of MM are associated with a poorer prognosis and decreased survival rates, with few remaining therapeutic options. Their cross-sectional dimension and the assessment of growth or shrinkage with either technique are two aspects included in the standard IMWG response criteria defining a partial response and a minimal response [13]. FDG-PET-CT has alternatively become established for the evaluation of extraosseous disease, since it is more sensitive than CT due to the uptake of FDG in these lesions and because it yields morphological, biochemical and functional information [18, 25, 33].

Skeletal-related complications in MBD negatively affect survival [34]. Diffuse osteopenia and vertebral compression fractures, which are observed in up to 80% of patients, are typical complications of MM but are no longer considered diagnostic disease criteria [21, 35]. This is due mainly to the fact that it is sometimes difficult to establish the exact cause of the fracture by CT. As a quick, affordable, easily accessible and nearly ubiquitously available imaging technique, WBCT is the modality of choice to assess spinal stability in cases with pathological neoplastic or osteoporotic fractures and in acute trauma or staging examinations. This assessment via CT is crucial, especially since 11–24% of patients with MM experience spinal cord compression during the course of their disease.

### Radiation dose in WBLDCT

Because of the high cumulative radiation dose from standard CT scans, WBLDCT has become the modality of choice for staging. Currently, the effective radiation dose is dependent on the type of CT scanner, and results from either lower kilovoltage peak (kVp) or lower tube current—time product (mAs) or a combination of both parameters. In 2008 we performed the first comparative studies on WBLDCT and WBMRI, where we used a multidetector CT scanner with 16 or 64 detector rows, a constant tube voltage of 120 kV and a tube current–time product of 100 mAs [11]. In 2005, Horger et al. presented a scan protocol calculated at a tube current–time product of 40 mAs with an effective radiation dose of a 4.1 mSv and 120 kV that was only 1.7-fold higher than the average radiation dose of XR (2.4 mSv) [36]. Technical advancements including automated modulation of the tube current and novel reconstruction algorithms have further improved the image quality and simultaneously reduced the effective radiation dose [37, 38].

A recent study examining 74 patients with various plasma cell diseases revealed that iterative reconstruction algorithms enabled WBLDCT scans with almost identical radiation exposure (2.7 ± 0,9 vs. 2.5 ± 0,9 mSv; *P* = 0,054), but superior detectability of bone lesions, skeletal fractures and vertebral compressions, in comparison to WBXR [39]. In 2018, the IMWG bone working group recommended the application of iterative reconstruction algorithms, if available, in order to reduce the image noise and streak artifacts of the WBLDCT images [17].

Dose reduction while maintaining the same image quality can alternatively be achieved by spectral shaping in WBLDCT [40]. Supplemental to the standard aluminum bowtie filter, spectral shaping uses an additional tin filter between the CT tube and the patient in order to absorb low-energy photons, which are less pertinent in high-contrast imaging of the lung or the bones. The corresponding retrospective case–control study compared the WB scan protocols of a third- and second-generation dual-source CT scanner at 100 kV in a total of 60 individuals, and resulted in a reduction of the mean effective dose of 74%. A recently published study investigating 30 patients using spectral shaping and a third-generation dual-source multidetector CT scanner further improved the effective dose [41]. Other improvements in dose reduction may be achieved by deep convolutional neural networks that are trained to remove CT image noise in order to improve the image quality of ultralow-dose CT scans to appear similar to normal-dose CT scans [42]. A 3D convolutional neural network was recently introduced with specific loss functions for QCT noise reduction to compute microstructural parameters such as tissue mineral density and bone volume ratio. This method allows the assessment of the 3D bone microstructure and has the potential to detect rarefaction of the trabecular network due to osteoporosis or other bone diseases [43]. It may further discriminate patients with and without vertebral fractures or indicate early stages of osteolytic processes that are not yet visible [44].

After all, further dose reduction of WBLDCT is preferable either way, considering the increasing relevance of surveillance imaging in patients suffering from MGUS and SMM.

Another technical achievement is DECT. Three different studies have shown that these calculated VNCa images allow the identification of plasma cell infiltration via color-coded maps and enable their quantification using region of interest (ROI)-based HU measurements [45–47]. A separate article in this issue addresses this topic.

### Positioning and technical parameters of WBLDCT

As described above, there are several options for producing CT images of reasonable diagnostic quality while maintaining acceptable radiation doses. In 2018, the IMWG bone working group published specific recommendations for the acquisition of WBLDCT in MM [17, 25].

No specific patient preparation and no oral or intravenous contrast administration is required for the acquisition of bone images. The patient lies supine on the CT table, and in order to enable precise evaluation, the entire skull, both humeri and both proximal tibia metaphyses should be included in the field of view. Therefore, both arms can be placed aside and above the head, with as little bending of the elbows as possible in order to avoid truncation artifacts. Alternatively, they can be put alongside the body, but underlaid with custom-made arm cushions that lift them above the level of the thoracic and lumbar spine in order to avoid beam-hardening artifacts. The arms of obese patients may also be placed in front of the body with both hands folded in order to maintain an elevated position and to shift the beam-hardening artifacts anteriorly [17].

**Fig. 2:**
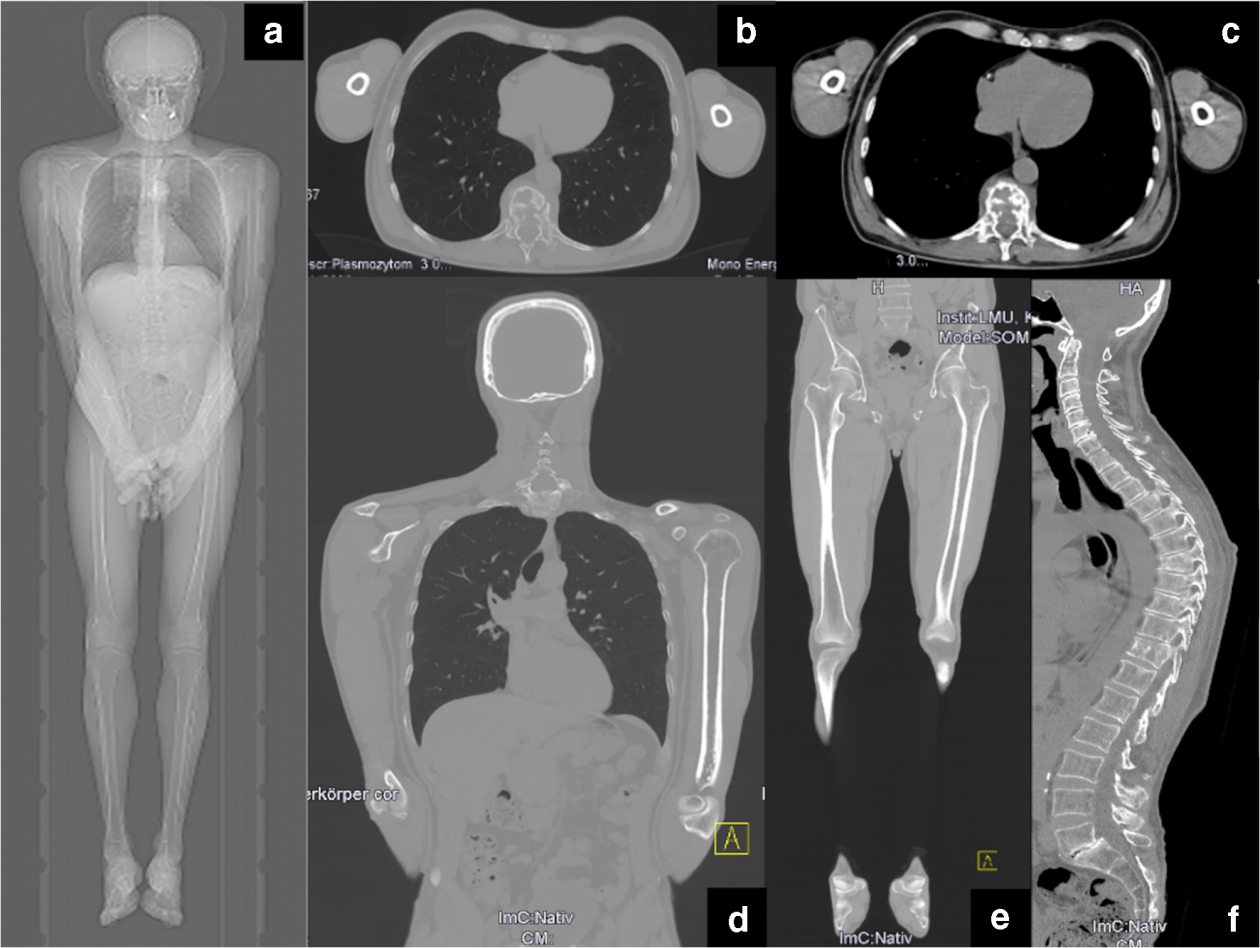
Image examples of a whole-body low-dose CT scan (**a**: scout). Images were reconstructed in axial 3 mm slices using the intermediate (**b**) and soft-tissue kernel (**c**) in order to evaluate soft-tissue masses and the intrathoracic and intra-abdominal organs. The skeleton is further reconstructed in coronal 3 mm slices using the bone kernel for the reading of the upper (skull, thorax, arms; **b**, *d*) and the lower parts (pelvis, legs; **e**) of the body. The spine is additionally reconstructed in sagittal orientation (**f**).

A multidetector CT scanner with at least 16 detector rows is required for WBLDCT in MM to ensure short scan times and to acquire thin slices while maintaining low cumulative radiation doses. As there are various data but no validated evidence for the optimal combination of tube voltage and tube current–time product, the IMWG bone working group recommends the application of 120 kV combined with 50–70 mAs and a collimation range of 0.5–1.5 mm. Image reconstruction should utilize a sharp, high-spatial-frequency convolution kernel to ensure increased spatial resolution enabling an optimal assessment of even small osteolytic lesions. Additional image reconstruction using a smooth convolution kernel ensures the optimal assessment of para- and extramedullary soft-tissue structures and enables the detection and attenuation measurement of medullary plasma cell infiltrates in the appendicular skeleton. Thin axial slices with an increment of 50% enable the detection of small osteolytic lesions and may serve as source images for multiplanar reformations (Fig. 2). Table 1 summarizes the abovementioned recommended technical parameters and includes accepted alternatives. As a matter of course, periodic calibration of the CT scanners ensures uniformity of the attenuation measurements over a period of time.

**Table 1:** Summary of the recommended technical parameters and accepted alternatives for WBLDCT in MM [17]

Parameters	Recommendation	Alternative
No. of detector rows	At least 16	
Field of view	From the cranial vertex to the prox. metaphyses of both tibiae incl. both humeri	
Regular patient positioning	Supine with both arms aside and above the skull with as little bending of the elbows as possible	Supine with both arms alongside the body underlaid with armrests to lift them above spine level
Positioning of obese or PRM	Supine with both arms placed in front of the body with both hands folded	
Tube voltage (kV)	120	140
Tube current–time product (mAs)	50–70	14–25
Collimation	0.5–1.5 mm	–
Reconstruction convolution kernel	Combination of a sharp, high-frequency “bone” kernel and a smoother “soft-tissue” kernel	One singular intermediate, middle-frequency kernel permitting acquisition of all images with different window settings*
Iterative reconstruction algorithms	Requested, if possible, to reduce image noise and artifacts while reducing radiation exposure	
Thickness/increment of axial slices	2/1 mm	3/1.5 mm
MPRs	requested in sagittal and coronal direction and parallel to the long axis of the prox. extremities	

Abbreviations: No.: number; prox.: proximal; incl.: inclusive; PRM: persons with reduced mobility; kV: kilovoltage; mAs: milliampere-seconds; mm: millimeters; MPRs: multiplanar reconstructions

* With the limitation that attenuation measurements of peripheral intramedullary lesions should not be performed on bone kernel-reconstructed images that were set on the soft-tissue window, but on soft-tissue kernel-reconstructed images

## Whole-body magnetic resonance imaging (WBMRI)

### Modality overview

**Fig. 3:**
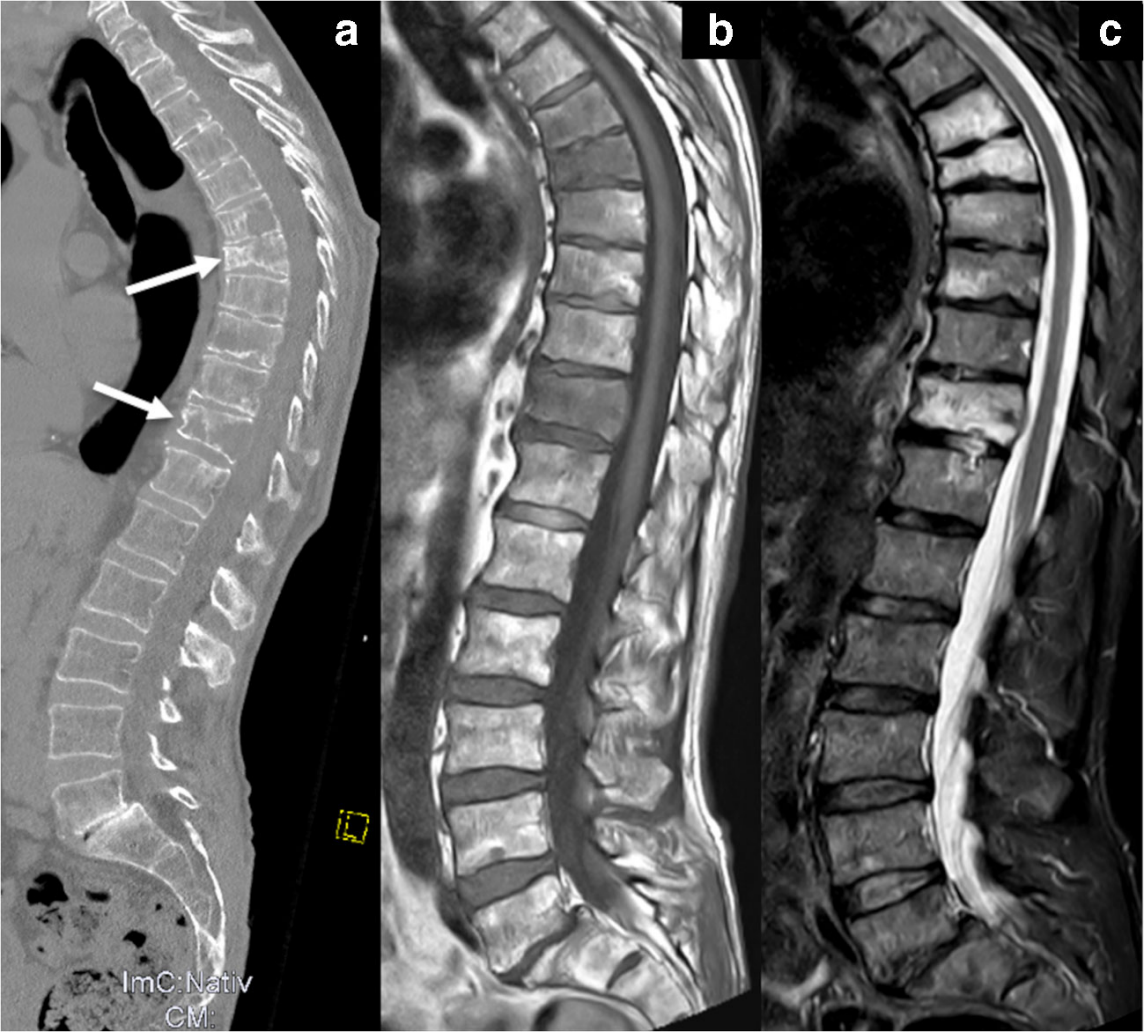
(**a**) Marked osteolytic lesions are detected in the 6th, 7th and 11th thoracic vertebral body and pathological fractures in the 7th and 11th vertebrae in the sagittal CT reconstruction of the spine in the bone window. Additional lesions are suspected in the 8th–10th vertebral body. MRI of the patient using a T1-weighted (**b**) and a STIR (**c**) sequence in sagittal orientation confirms the involvement of myeloma in the 6th, 7th and 11th vertebrae, while end plate-associated edema due to degenerative disease is seen in the 8th and 9th thoracic vertebrae. Thus, CT can sometimes over-diagnose myeloma involvement in cases of osteoporosis and inhomogeneous bone structure

The major advantage of MRI in comparison to other cross-sectional imaging techniques is its superior soft-tissue contrast, offering the opportunity to visualize bone marrow infiltration before destruction of the mineralized bone tissue occurs [21]. MRI is considered to be the most sensitive and most specific imaging modality for noninvasive detection of early plasma cell invasion of the bone marrow, because it is able to evaluate medullary composition and cellularity [48]. Specific MRI findings may therefore prompt the upgrading of patients from precursor stages to active MM, even though the corresponding WBLDCT does not yet depict osteolysis (Fig. 3) [11, 49]. In addition, diffusion-weighted imaging (DWI) in WBMRI offers a functional imaging modality for the evaluation of the medullary bone, providing excellent accuracy for the differentiation of pathological from normal bone marrow. A meta-analysis from 2018 reported a pooled sensitivity of 89% and a pooled specificity of 87% of the ADC, respectively, for the differentiation of benign and malignant vertebral bone marrow lesions [50].

In 2014, MRI findings were included in the diagnostic criteria of the IMWG. Since 2015, the IMWG recommends MRI as the gold standard for the imaging of the axial skeleton, the assessment of dolorous lesions and the differentiation of benign from malignant osteoporotic vertebral fractures [48]. Additionally, it is considered the method of choice to for the assessment of painful complications and spinal cord compression. Since 2016, the IMWG guidelines consider the detection of one or more focal lesions at least 5 mm in size on MRI as a criterion for active MM. Similar to the recommendations for WBLDCT, the finding of equivocal or smaller lesions on MRI should not be over-interpreted but should prompt a follow-up examination within 3–6 months in order to verify or exclude active MM. In contrast to the evidence of focal lesions as mentioned above, a diffuse infiltration pattern of the bone marrow on MRI is not yet considered a myeloma-defining event. According to the IMWG guidelines, it should prompt surveillance by follow-up imaging within 3–6 months, or MM may be confirmed with biopsy and histological evidence of ≥60% of plasma cells in the bone marrow specimen [25, 51].

Systematic WBMRI or alternatively MRI of the spine and the pelvis are further recommended by the IMWG as part of the initial assessment of asymptomatic patients and patients suffering from SMM [48, 52]. Focal lesions and diffuse medullary abnormalities are considered as suspect imaging features in these cases and are associated with a higher risk of progression to active MM [6]. They can be identified by MRI during the initial stage of disease, when osteolytic destruction is not yet evident on WBLDCT. Focal cortical or trabecular lesions at least 5 mm in diameter on MRI should be considered as criterion for symptomatic disease and initiation of therapy. A follow-up MRI after 3–6 months is further recommended for patients with SMM exhibiting equivocal focal lesions, as growth dynamics have additional predictive value, and SMM patients with progression, indicated by an increase in number or size of focal lesions, should be considered as symptomatic and requiring therapy [6, 48]. If MRI is unavailable, [^18^F]-FDG PET/CT is the method of choice to distinguish between SMM and active MM [18].

Systematic WBMRI is also proposed for the workup of patients with solitary bone plasmacytoma, while [^18^F]-FDG PET/CT is recommended in cases of suspected solitary extramedullary plasmacytoma [5, 53]. Solitary bone plasmacytoma is twice as prevalent and associated with a higher risk of progression to MM within the first 3 years [54]. The most important purpose of a WBMRI examination in solitary extramedullary plasmacytoma is the exclusion of additional bone marrow lesions and/or further soft-tissue masses, which differentiates this disease from systemic MM [25]. A second aspect is the detectability of a diffuse bone marrow infiltration, which may prompt further examinations and consequently lead to the upstaging of patients and modified treatment strategies [51]. Yearly follow-up examinations using the same imaging technique are recommended for the first 5 years after the initial diagnosis of solitary bone plasmacytoma.

In contrast, WBLDCT is recommended as first-line imaging modality in suspected high-risk non-IgM MGUS to rule out MM [25]. WBMRI is an accepted alternative if WBLDCT is not available and is helpful in the assessment of benign vertebral lesions, bone marrow edema and fractures in elderly patients. Furthermore, WBMRI or alternatively MRI of the spine and the pelvis are suggested in patients with equivocal findings on WBLDCT and suspected development of MM but no other apparent clinical evidence of myeloma such as hypercalcemia, renal failure, anemia and bone lesions, so called CRAB criteria [5].

### Imaging findings in WBMRI

Imaging of bone marrow lesions in MM patients is inhomogeneous and differentiates between five patterns of plasma cell infiltration. They include (1) a normal appearance of the bone marrow despite minor microscopic plasma cell clusters, (2) focal lesions, which are defined as lesions of at least 5 mm in diameter, (3) a homogeneous diffuse infiltration, (4) a mixture of focal lesions and diffuse infiltration, and (5) a variegated or “salt-and-pepper” pattern due to an inhomogeneous bone marrow appearance with interposition of fat islands [10, 48, 55, 56]. A high tumor burden is typically associated with a diffuse pattern or with focal lesions, while a normal or variegated MRI pattern is seen in patients with a low tumor burden [57]. Nevertheless, the IMWG has not yet defined the diffuse pattern as a criterion for symptomatic disease [48].

**Fig. 4:**
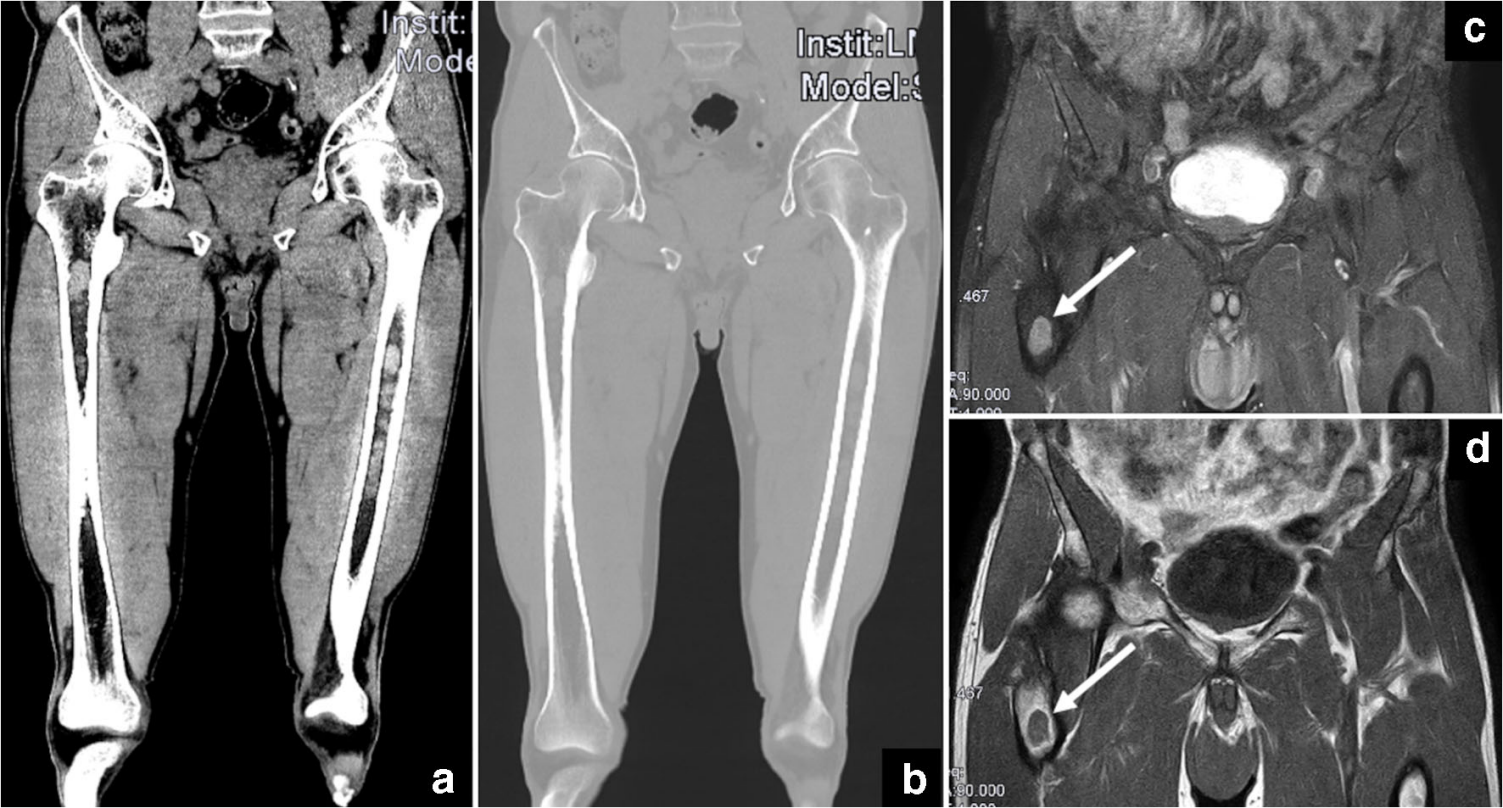
Illustration of typical marrow infiltrates in both femurs. (**a**) Coronal CT reformation in the soft-tissue window setting shows focal lesions with high signal density (75 HU) within the normal fatty marrow (black arrows). (**b**) The lesions can be missed in the corresponding CT reformation in the bone window setting. (**c**, **d**) The focal lesions exhibit a hypointense signal in the corresponding coronal T1w MRI scan and a hyperintense signal in the T1w fat-saturated scan (white arrows)

T1w SE sequences are the method of choice for the morphological evaluation of bone marrow, as suspicious, hypointense lesions contrast with the medullary fat that increases with increasing age. The lesions are usually as low as muscle signal intensity, whereas marrow edema is usually less hypointense. Sequences with fat suppression confirm suspicious lesions, which show a high signal intensity due to their high cellularity and water content. T2-weighted (T2w) SE sequences optimally visualize the spinal canal and enable the detection of bone and soft-tissue edema. In contrast to spectral fat saturation techniques, frequency-selective fast saturation techniques, such as short-tau inversion recovery (STIR), produce more homogeneous fat saturation in large fields of view. This is important for a whole-body setting (Fig. 4).

On DWI, signal intensity is high, with a low ADC, due to restriction of water protons in tumor nodules. Focal lesions enhance after the intravenous application of gadolinium. Suspicious focal bone lesions are easier to detect in elderly patients because of their higher medullary content of fat. It is important to realize that a focal bone marrow lesion on MRI is not automatically equal to an osteolytic lesion with bone destruction on CT. Focal medullary lesions on MRI simply illustrate a dense plasma cell infiltration that may be associated with a lytic lesion or that may precede bone destruction and the development of an osteolytic lesion. Thus, both imaging modalities, CT and MRI, provide complementary information and should be applied accordingly [5]. Corresponding guidelines for the implementation, acquisition and interpretation of WBMRI and WBLDCT have been developed [17, 25, 51].

**Fig. 5:**
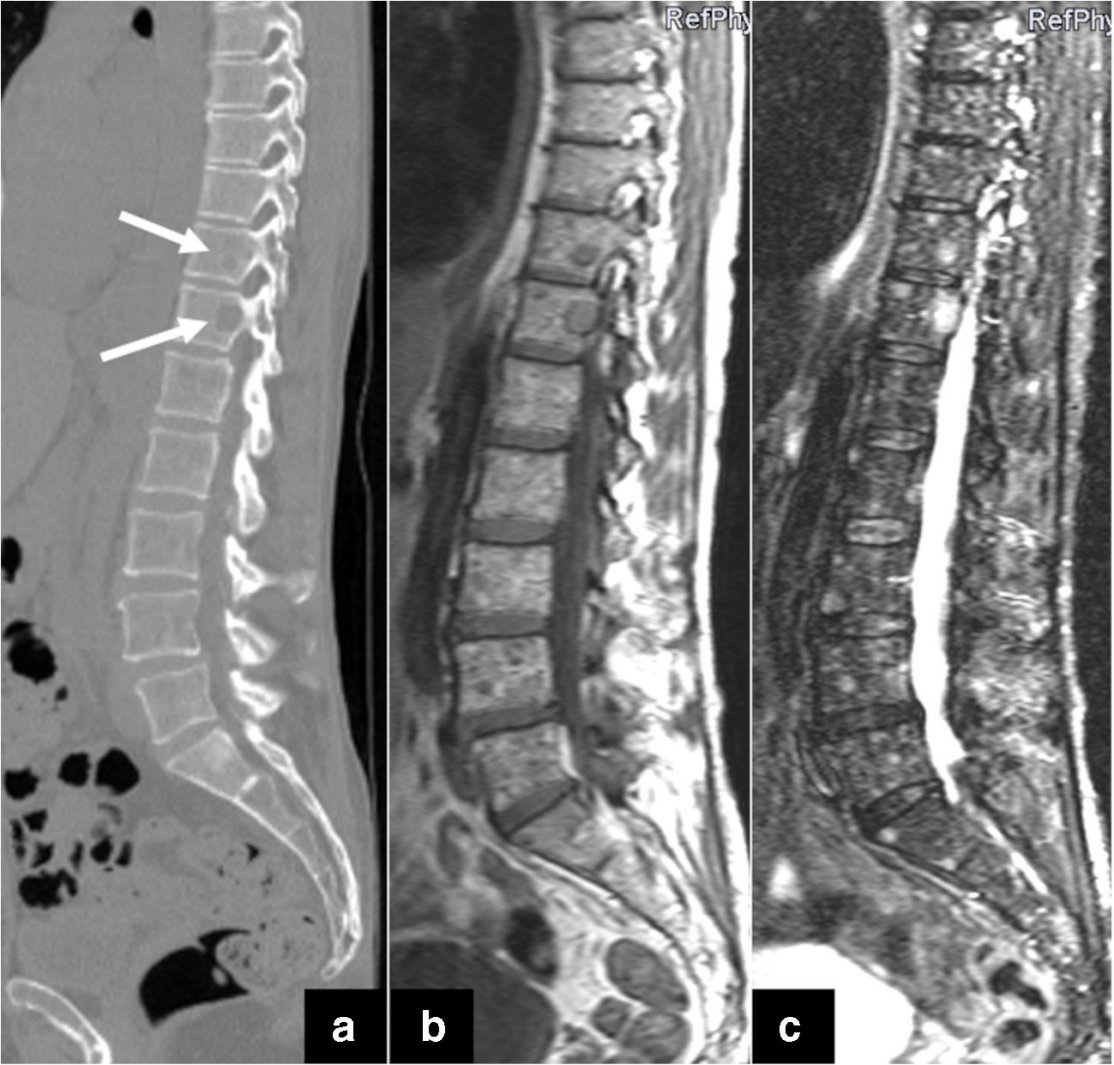
CT often underestimates tumor mass because on CT only myeloma infiltrates which have already led to osteolytic lesions can be detected. (**a**) The white arrows mark two osteolytic lesions in the 11th and 12th vertebral body on the sagittal CT reformation. The corresponding sagittal T1w MRI scan (**b**) and the STIR sequence (**c**) illustrate that every vertebral body is affected by myeloma

**Fig. 6:**
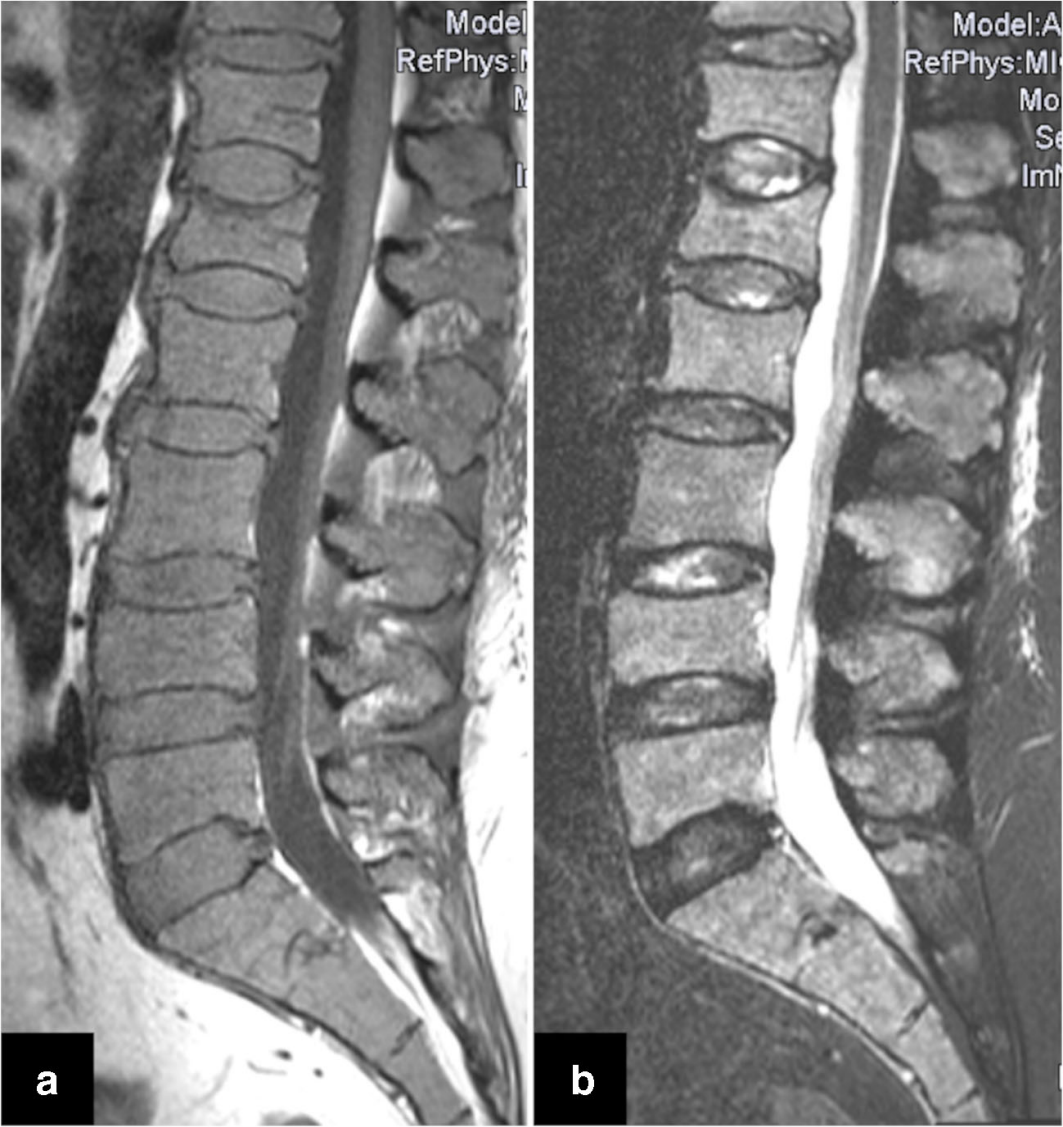
MRI illustration of a diffuse medullary infiltration pattern in myeloma using T1w (**a**) and STIR (**b**) sequences. It is characterized by decreased signal intensity of the bone marrow on T1w sequences (**a**) and a correspondingly increased signal on STIR (**b**) sequences. On T1w images (**a**), the signal intensity of the bone marrow is equal to the intervertebral disc and the muscle, which is known as the “bright disc” sign

Conversely, the detection of diffuse marrow infiltration sometimes remains challenging (Fig. 5). A diffuse medullary infiltration is characterized by a decreased signal on T1w sequences and a correspondingly increased signal on fat-saturated sequences. Low-grade diffuse disease (< 20 vol%) usually cannot be detected with MRI. In these initial stages, the fat component is often increased. Later, adipocytes are replaced by plasma cells, leading to a reduction in signal intensity on T1w SE images. T1w images have proven to be the most sensitive sequence for the evaluation of diffuse disease [29]. In intermediate-grade diffuse bone marrow infiltration, the signal is reduced on T1w turbo spin-echo (TSE) images and eventually slightly increased on fat-saturated sequences. In high-grade plasmacytosis (> 50 vol%), the signal is markedly decreased on T1w TSE images. The signal intensity of the marrow is almost the same as the intervertebral disc or as low as muscle. This was also described as the “bright disc” sign (Fig. 6) [29, 58, 59].

Contrast application can help in distinguishing normal from abnormal marrow, as contrast enhancement is higher in myeloma due to the high neovascularisation, at least in intermediate- and high-grade plasmacytosis [60, 61]. Red marrow reconversion has to be mentioned as a differential diagnosis in diffuse hypercellularity. It has been described early after initiation of chemotherapy, after administration of bone marrow-stimulating factors like erythropoietin or granulocyte colony-stimulating factor [14]. A reduced signal intensity in all MRI sequences due to medullary iron overload is another phenomenon that is frequently seen after stem cell transplantation or repeated blood transfusions, and which should be considered in patients suffering from an advanced stage of MM. However, here, the signal on T2w or fat-saturated sequences is also low [51].

In contrast to WBLDCT, MRI is primarily applied for neuroimaging in extraosseous MM [24, 62, 63]. MRI is considered the modality of choice when extraosseous soft-tissue masses are located in paraspinal or paravertebral areas, and it is the first method of choice whenever spinal cord compression is suspected [48, 64]. Because of the brain–blood barrier, direct involvement of the central nervous system (CNS) is very rare, with an incidence of about 1% in MM patients. It usually develops during disease relapse or progression, either secondarily due to an expanding bone lesion or primarily by hematogenous spread or via meningeal myelomatosis [63, 65, 66]. Unenhanced and contrast-enhanced MRI is recommended in cases with neurological symptoms suggesting CNS manifestations, but image findings are nonspecific, and important differential diagnoses are drug toxicity and metabolic alterations [65].

The liver, spleen and lymph nodes are considered the most frequently affected abdominal sites in extraosseous MM. Due to the high soft-tissue contrast, MRI outperforms CT in the detection of a diffuse plasma cell infiltration of the parenchymatous abdominal organs, which is typically associated with hepato- or splenomegaly and is reported to be the most common pattern of manifestation on autopsy studies [32].

Recently, the incidence of CNS and extraosseous manifestations in patients suffering from MM seem to be increasing, perhaps due to modern therapies and more sensitive imaging techniques [67]. Both manifestation patterns are associated with poorer survival rates and prognosis, while the response to conventional treatment strategies remains poor [63, 67].

Apart from that, MRI is also able to reliably diagnose an avascular epiphyseal necrosis, which is a frequent incidental finding and complication of myeloma treatment. Functional and morphological MR imaging of the bone marrow microstructure and composition thereby enables the detection of preclinical ischemic stages identifying patients at risk prior to manifest bone infarcts [68]. This may reduce patients’ distress and therapy-associated costs [68, 69].

### Advances in functional WBMRI

#### Diffusion-weighted MRI (DWI-MRI)

Diffusion-weighted imaging (DWI) in WBMRI adds functional information to the morphological sequences. It is the most sensitive MRI sequence for the detection of metastatic bone lesions [53, 70] and it is quick to perform and interpret, and enables objective measurements [51, 53]. As the ADC value is directly related to the tissue cell density, the signal intensity of the bone marrow on DWI and ADC maps during the course of the disease is associated with the amount of medullary plasma cell infiltration [64, 71].

DWI-MRI is superior to conventional sequences in the detection of spinal medullary lesions and is reliably able to differentiate between MM, SMM and MGUS [72]. Nevertheless, DWI-MRI lacks specificity when its findings are interpreted in isolation [51]. The technique is addressed in detail in a separate article in this special issue.

#### Dynamic contrast-enhanced MRI (DCE-MRI)

Dynamic contrast-enhanced MR imaging (DCE-MRI) is another advanced, functional technique that can be used for noninvasive evaluation of tumoral neoangiogenesis, perfusion characteristics, and tissue composition and permeability. It is based on a computer-assisted analysis of the distribution of gadolinium within the intra- and extracellular matrix in sequential images. Using ROIs, the changing patterns that occur during the passage of the contrast bolus are transformed into semiquantitative parameters that are plotted and expressed as time–intensity curves, which correlate with histological findings in MM. Additionally, a quantitative analysis can be performed using a pharmacokinetic mathematical model [71, 73–75]. At present, DCE-MRI is not recommended for the evaluation of bone marrow disease, but it may provide useful information about treated lesions and may be useful in the context of clinical trials investigating novel agents.

Five different types of time–intensity curves have been described in the literature. Medullary infiltration in MM is typically characterized by type 4 curves, which display a quick and steep wash-in with a rapid wash-out. Type 3 curves, which depict a quick and steep wash-in with a wash-out plateau, and type 5 curves, which represent a quick, but less intense wash-in and late stable or increasing enhancement, are less frequently seen in MM. Type 1 and 2 curves, which illustrate either no or a slow enhancement pattern, are characteristic for normal yellow bone marrow or are typically observed in patients with MGUS [74]. One study by Delorme et al. was able to show that even in low-grade diffuse disease and SMM, perfusion is increased in time-resolved contrast-enhanced images when compared to healthy controls [76, 77].

DCE-MR images are acquired during and immediately after the intravenous administration of a bolus of contrast medium (0.2 ml/kg) at an injection rate of 5 ml/s. Fast or ultrafast MRI sequences, such as gradient-echo (GRE) sequences with very short repetition and echo times, are recommended for the evaluation of early-contrast-enhancement kinetics, because they combine rapid sequential acquisition of 1–3 s per image with a reasonable temporal and spatial resolution. The fat-suppressed T1w ultrafast GRE sequence of the thoracolumbar spine is usually followed by static fat-suppressed T1w images [64, 71].

### Chemical-shift MRI

Chemical-shift-based fat-water separation imaging using T1w GRE sequences is able to detect visceral and medullary fat deposits on in-phase images and may be used to calculate fat-fractioning images [78]. Therefore, the technique enables the detection of microscopic fat deposits within the bone marrow and the visceral organs because medullary fat and reservoirs of adipocytes, e.g., within the liver, exhibit a high signal intensity on in-phase images and a low signal on opposed-phase images. Moreover, chemical-shift imaging can be used to calculate fat-fraction images and opposed-phase images can aid the characterization of bone lesions [51]. In contrast to hemangiomas, which contain fat and thus are hyperintense on fat-selective images but hypointense on water-selective images, MM manifestations exhibit the opposite signal characteristics due to their high cellularity and water content.

Functional chemical-shift MRI sequences are useful in younger patients with medullary plasma cell infiltration but simultaneously a high content of hematopoietic bone marrow, and in cases with red marrow reconversion, during therapy because pathological conditions exhibit a signal decrease of more than 20% at opposed-phase examinations in comparison to in-phase MRI. In addition, functional chemical-shift MRI sequences are able to indicate medullary plasma cell infiltrates as low as 10% during the initial stage of disease, while conventional MRI sequences depict a normal bone marrow pattern [21, 79].

### Positioning and technical parameters of WBMRI

As described above, there are several options for morphological and functional WB-MRI. In 2019, a multidisciplinary international expert group published current guidelines for the acquisition, interpretation and reporting of WBMRI in MM in order to standardize the diagnosis and to enable accurate assessment of the treatment response using the Myeloma Response Assessment and Diagnosis System (MY-RADS) [51].

The consensus for the technical prerequisites is that a field strength of 1.5 T should be used for WBMRI as a standard for imaging of MM due to its robustness and widespread availability [51]. Whenever possible, follow-up examinations should be performed on a similar unit. No specific patient preparation and no oral contrast administration is required for the acquisition of bone images. The patient lies supine on the MRI table in a headfirst position, and the head should rest in an appropriate head coil, while both arms should be positioned by the sides. Surface coils are recommended to maximize the signal-to-noise ratio of images, and they should cover the patient from the skull vertex to the mid-thigh without any gaps in between, to enable a precise evaluation. Wide anatomical coverage including the rib cage, the shoulder articulations and the long bones of the extremities is essential, as up to 50% of lesions may be missed by imaging the spine alone [80]. As WBMRI requires rather long scan times, attention should be paid to patient comfort and the most relevant sequences should be acquired first, since the risk of motion artifacts rises with increasing scan time. A general quality control program for MRI and periodic validation of ADC and fat fraction measurements is mandatory.

Studies should always include a combination of two morphological sequences (T1w and STIR) and at least one functional imaging sequence. The most important functional imaging sequence of choice is DWI, which should be included in every study, while DCE-MRI and chemical-shift MRI sequences are secondary to that. The recommended core clinical protocol should take about 30 min, and is designed for the detection of the disease. The comprehensive protocol is required for the examination of soft tissue, extramedullary disease and patients with periodic tumor response assessments. It takes about 45–50 min. Detailed information about the imaging protocol is provided in Table 2, and a summary of the imaging recommendations of the IMWG is provided in Tables 3 and 4.

**Table 2:** Summary of the recommended technical parameters and accepted alternatives for the WBMRI examination in MM [51]

Sequence	Orientation	FOV	Section thickness/ technical details	Aim/ target structure	Protocol
1) T1w SE or TSE	Sagittal	wh.-sp.	4–5 mm	Bone marrow	*, #
2) STIR or T2w SE fs or T2w	Sagittal	wh.-sp.	4–5 mm	Spinal canal, bone/soft-tissue edema	*, #
3) T1w gradient-echo or T1w TSE	Sagittal/axial	wh.-bd.	5 mm or thin, isotropic	Fat fraction maps	*, #
4) DWI SE-EPI, ADC calc., 3D MIP recon. of highest b-value images	Axial	wh.-bd.	5 mm contiguous sectioning/2 b-values (b50–100, b800–1000 s/mm^2^)	Bone marrow	*, # (additional b500–600 s/mm^2^)
5) T2w TSE	Axial	wh.-bd.	5 mm contiguous sectioning/multiple stations, preferably matching DWI		*optional, #
6) Local assessment	Individual			Symptomatic/known sites outside standard FOV	#optional

Abbreviations: FOV: field of view; T1w: T1-weighted; SE: spin-echo; TSE: turbo spin-echo; wh.-sp.: whole spine; mm: millimeters; T2w: T2-weighted; GRE: gradient-echo; wh.-bd.: whole-body = vertex to knees; DWI: diffusion-weighted imaging; EPI: echo-planar imaging; calc.: calculation; ADC: apparent diffusion coefficient; MIP: maximum intensity projection; recon.: reconstruction; s: seconds; 3D: 3-dimensional; STIR: short tau inversion-recovery; fs: fat-saturated

° STIR or T2w FS preferred over T2w TSE, due to higher signal contrast

* Recommended as part of the core clinical protocol

**#** Recommended as part of the comprehensive protocol

**Table 3:** Summary of the imaging recommendations of the IMWG [25] to establish the initial diagnosis and for the follow-up assessment of PCDs

	WBLDCT	WBMRI or MRI of the spine and pelvis
Imaging recommendations to establish the initial diagnosis		
MGUS	To exclude MM in suspected high-risk non-IgM MGUS^1^	- Alternative, if CT is unavailable - Next step to evaluate equivocal findings of CT
SMM	Method of choice to detect osteolytic lesions**	To exclude focal lesions as myeloma-defining events, if CT is negative^2^
Solitary plasmacytoma	–	In solitary bone plasmacytoma^3^
MM	Method of choice to detect and evaluate the extend of osteolytic lesions^4^	Next step to exclude focal lesions as myeloma-defining events, if CT is negative and no other myeloma-defining event is present^5^
Imaging recommendations for follow-up examinations and the assessment of treatment response		
MGUS	Not indicated unless signs of progression to symptomatic disease occur	
SMM	- Repetition of same technique used at initial diagnosis at yearly intervals for at least 5 years depending on the risk factors - Additional alternating WBLDCT in cases with a high risk of progression to identify small osteolytic lesions	
Solitary plasmacytoma	Repetition of same technique used at initial diagnosis at yearly intervals for at least 5 years	
MM	- Repetition of same technique used at initial diagnosis to provide comparability - Follow-up imaging should be adapted in cases of progression, when the repeated and initially applied imaging technique does not reveal post-treatment imaging results that justify a change of treatment - WBLDCT when a relapse is suspected to evaluate the extent and dynamics of bone destruction as the most clinically relevant parameter^6^	

Abbreviations: WBLDCT: whole-body low-dose computed tomography; WBMRI: whole-body magnetic resonance imaging; MGUS: monoclonal gammopathy of undetermined significance is a plasma cell dyscrasia in which plasma cells or other types of antibody-producing cells secrete a myeloma protein, i.e., an abnormal antibody, into the blood; this abnormal protein is usually found during standard laboratory blood or urine tests; SMM: smoldering multiple myeloma (also sometimes known as asymptomatic myeloma) is an early form of myeloma, which usually progresses to active myeloma, but at a slow rate. In smoldering myeloma, abnormal cells can be detected in the bone marrow, and abnormal protein can be detected in the blood and/or urine; MM: multiple myeloma

^1^ Additional PET/CT if WBLDCT is positive

^2^ PET/CT can be used as an alternative to WBLDCT and instead of WBMRI if the latter is unavailable or in cases with specific contraindications

^3^ PET/CT in patients with solitary extramedullary plasmacytoma and as an alternative in solitary bone plasmacytoma, if MRI is unavailable

^4^ PET/CT can be used as an alternative to WBLDCT

^5^ PET/CT can be used as an alternative to MRI if the latter is unavailable or in cases with specific contraindications, and it is the preferred imaging method to generate a baseline for follow-up assessments

^6^ Yearly follow-ups are recommended for patients with residual lesions detected by PET/CT because of the high risk of early progression

**Table 4:** Summary and comparison of the main features, advantages and limitations of CT and MRI in MM and other PCDs

	CT	MRI
Main features	- Morphological imaging in clinical routine, functional imaging (DECT) only in study settings - Highest sensitivity for the detection of osteolytic bone lesions - Visualization of bone marrow infiltration in the extremities - Gold standard for the assessment of spinal stability in vertebral fractures - Necessary/supportive for the planning of radiotherapy, surgeries and biopsies - Valuable for the assessment of treatment response and relapse during follow-up to evaluate the extent and dynamics of bone destruction as the most clinically relevant parameter	- Morphological and functional imaging (DWI) in clinical routine - Additional functional imaging options (DCE, chemical shift with fat fraction maps) in study settings - Highest sensitivity for the detection of bone marrow infiltration - Depiction of medullary infiltration pattern - Depiction of non-osteolytic bone marrow infiltration prior to bone destruction - Gold standard for the assessment of spinal cord and nerve root compression - Gold standard for the differentiation of benign and malignant vertebral fractures - Prognostic relevance - Possible value for the assessment of treatment response and relapse during follow-up when functional imaging (DWI) is applied
Technical advantages	- Better availability - Shorter acquisition time - Higher patient comfort - Lower costs - No administration of contrast agents required for evaluation of MBD	No radiation exposure
Technical limitations	- Radiation exposure	- Limited availability - Longer acquisition time - Patient discomfort, may be significant in patients with claustrophobia, or older metal implants
Administration of contrast agents	- Adverse impact on kidney function in patients with preexistent renal impairment - Possible allergic reactions - Possible thyrotoxicosis	- Adverse impact on kidney function in patients with preexistent renal impairment - Disassociation and deposition of linear GBCAs

Abbreviations: CT: computed tomography; MRI: magnetic resonance imaging; DECT: dual-energy computed tomography; DWI: diffusion-weighted imaging; DCE: dynamic contrast-enhanced; GBCAs: gadolinium-based contrast agents

## Limitations of WBMRI

Restrictions that have to be addressed include the higher cost but lower availability of WBMRI when compared with WBLDCT. The more time-consuming WB examination may be challenging for patients with active MM and MBD who suffer from bone pain, as they are asked to lie as motionless as possible during image acquisition. Otherwise, motion artifacts may impede the interpretation of MR images. Claustrophobia is an additional serious limitation, which may be worsened by the application of body matrix anterior coils or the psychological aftereffects of the serious cancer diagnosis and rather intensive therapeutic treatments such as chemotherapy or stem cell transplantation. Although the number MRI-compatible metallic implants and medical devices is increasing, the technique is still contraindicated in patients with several older or incompatible models, due to the risk of device failure and heating.

Additionally, renal impairment is a frequent complication of myeloma, and restricted renal function is typically associated with a higher risk of complications in patients receiving MRI-contrast agents. A prospective trial by Hillengass et al. revealed no prognostic impact of the administration of gadopentetate-dimeglumine on progression-free survival in patients suffering from PCDs, when the current guidelines for the application of MRI contrast agents were respected [81]. Nevertheless, a significant adverse impact on kidney function was seen in symptomatic myeloma patients, who already exhibited reduced renal parameters at baseline, while patients with normal or slight renal impairment recovered.

In this context, the currently investigated and frequently discussed phenomenon of gadolinium deposition after serial injections of linear gadolinium-based contrast agents (GBCAs) needs to be addressed. Clinical data regarding the deposition in extracranial tissues, especially in the bone and the bone marrow, are rare [82]. A recently published meta-analysis from preclinical and clinical pharmacokinetic data confirmed the effect of prolonged excretion of linear GBCAs and thereby indirectly demonstrated the existence of a deep compartment of distribution for gadolinium in the body [83]. This is especially true for linear GBCAs. However, no specific clinical symptoms possibly related to toxic effects of the accumulation have been observed so far, and no specific interference with the imaging findings in the bone during periodic staging MRI examinations have been reported thus far [82].

## Summary

Imaging in the diagnosis, follow-up and evaluation of complications in MM has evolved and improved tremendously over the past two decades.

WBLDCT has replaced the WBXR survey and enables the highly sensitive detection of osteolytic lesions in MBD. It is the first method of choice for morphological imaging of patients suffering from myeloma in clinical routine, and the gold standard for the assessment of spinal stability in MBD-associated vertebral fractures. The planning of surgical device implantation and the guiding of biopsies in focal lesions are further indications for a dedicated CT exam. The IMWG now recommends a WBLDCT examination in high-risk patients suffering from non-IgM MGUS and in SMM in order to exclude osteolytic bone destruction as features of active MM.

WBMRI is the alternative to WBLDCT, if CT is unavailable, and is the method of choice to evaluate equivocal findings of CT. WBMRI reliably visualizes early medullary plasma cell invasion before bone destruction. In MM-associated complications and extraosseous manifestations, MRI is the first method of choice in patients with suspected spinal cord compression, and DWI-MRI is considered the most sensitive technique for the differentiation of benign osteoporotic and malignant vertebral compression fractures. WBMRI is further recommended in SMM patients with negative or inconclusive CT results to rule out two or more focal lesions, which are considered a myeloma-defining event. Functional MRI provides information on lesion composition in active MM with consequent prognostic implications and offers a high potential for the assessment of treatment response.

The recommendations for the acquisition, interpretation and reporting of WBLDCT and the corresponding MY-RADS recommendations for MRI enable a standardized systemic application of both imaging modalities in clinical routine across various locations, thereby creating a fundamental cornerstone for multicenter trials.

## Abbreviations

ADC: Apparent diffusion coefficient
CNS: Central nervous system
CT: Computed tomography
DCE MRI: Dynamic contrast-enhanced magnetic resonance imaging
DECT: Dual-energy computed tomography
DWI-MRI: Diffusion-weighted imaging magnetic resonance imaging
[^18^F]-FDG: [Fluorine-18]-Fluorodeoxyglucose
GBCA: Gadolinium-based contrast agents
GRE: Gradient-echo
IMWG: International Myeloma Working Group
MBD: Myeloma-associated bone disease
MGUS: Monoclonal gammopathy of undetermined significance
MM: Multiple myeloma
MRI: Magnetic resonance imaging
MY-RADS: Myeloma Response Assessment and Diagnosis System
PCD: Plasma cell disorders
PET/CT: Positron emission tomography computed tomography
SE: Spin-echo
STIR: Short-tau inversion recovery
SMM: Smoldering multiple myeloma
T1w: T1-weighted
T2w: T2-weighted
TSE: Turbo spin-echo
VNCa images: Virtual non-calcium images
WBCT: Whole-body computed tomography
WBLDCT: Whole-body low dose computed tomography
WBMRI: Whole body magnetic resonance imaging
WBXR: Whole-body radiographic skeletal survey
XR: Conventional radiography
